# Phosphorylation of cyclophilin D at serine 191 regulates mitochondrial permeability transition pore opening and cell death after ischemia-reperfusion

**DOI:** 10.1038/s41419-020-02864-5

**Published:** 2020-08-19

**Authors:** Stephen Hurst, Fabrice Gonnot, Maya Dia, Claire Crola Da Silva, Ludovic Gomez, Shey-Shing Sheu

**Affiliations:** 1 Center for Translational Medicine, Department of Medicine, Sidney Kimmel Medical College, Thomas Jefferson University, Philadelphia, PA 19107 USA; 2 Univ Lyon, CarMeN Laboratory, INSERM, INRA, INSA Lyon, Université Claude Bernard Lyon 1, 69500 Bron, France

**Keywords:** Phosphorylation, Myocardial infarction

## Abstract

The mitochondrial permeability transition pore (mPTP) plays a critical role in the pathogenesis of cardiovascular diseases, including ischemia/reperfusion injury. Although the pore structure is still unresolved, the mechanism through which cyclophilin D (CypD) regulates mPTP opening is the subject of intensive studies. While post-translational modifications of CypD have been shown to modulate pore opening, specific phosphorylation sites of CypD have not yet been identified. We hypothesized here that phosphorylation of CypD on a serine residue controls mPTP opening and subsequent cell death at reperfusion. We combined in silico analysis with in vitro and genetic manipulations to determine potential CypD phosphorylation sites and their effect on mitochondrial function and cell death. Importantly, we developed an in vivo intramyocardial adenoviral strategy to assess the effect of the CypD phosphorylation event on infarct size. Our results show that although CypD can potentially be phosphorylated at multiple serine residues, only the phosphorylation status at S191 directly impacts the ability of CypD to regulate the mPTP. Protein-protein interaction strategies showed that the interaction between CypD and oligomycin sensitivity-conferring protein (OSCP) was reduced by 45% in the phosphoresistant S191A mutant, whereas it was increased by 48% in the phosphomimetic S191E mutant cells. As a result, the phosphoresistant CypD S191A mutant was protected against 18 h starvation whereas cell death was significantly increased in phosphomimetic S191E group, associated with mitochondrial respiration alteration and ROS production. As in vivo proof of concept, in S191A phosphoresistant rescued CypD-KO mice developed significantly smaller infarct as compared to WT whereas infarct size was drastically increased in S191E phosphomimetic rescued mice. We conclude that CypD phosphorylation at S191 residue leads to its binding to OSCP and thus sensitizes mPTP opening for the subsequent cell death.

## Introduction

During myocardial infarction, reperfusion injury represents a significant amount of the resultant irreversible damage^1,2^. Mitochondrial permeability transition pore (mPTP) opening is recognized to replay a crucial role in this phenomenon^3–5^. The identity of the current pore-forming protein(s) of mPTP is still debated^6^. Although recent publications show that mPTP formation does not require the c, b, or oligomycin sensitivity-conferring protein (OSCP) subunits of the ATP synthase^7^, other studies suggest that subunit c is the conducting core^8,9^, or the interface between two ATP synthases forms the conductive mPTP^10^. It cannot be excluded that in addition to a core‐conducting structure, several alternative structures exist as regulatory components.

The prolyl isomerase cyclophilin D (CypD) in the matrix is an important regulator of mPTP opening^10,11^. CypD was identified as a target of the cyclosporine A (CsA) drug, which inhibits mPTP opening^12^. In vitro, the addition of CsA inhibits mitochondrial swelling and increases mitochondrial calcium retention capacity (CRC)^13^, conferring protection against lethal stress. In vivo, the inhibition of CypD has been reported to reduce infarct size in preclinical studies^14–17^, but failed to be translated in human clinics^18,19^, highlighting the need for new CypD inhibitors. Additionally, the molecular mechanism of how CypD binds to the mPTP still remains unknown.

Significant progress has been made regarding the upstream regulation of the mPTP, particularly the post-translational modification of CypD and how it modifies pore sensitivity^20–24^. It was reported that Glycogen Synthase Kinase-3β (GSK3β) inhibitors prevent CypD phosphorylation, yet the phosphorylation site(s) was (were) not identified^25,26^. Indeed, the kinase GSK3β has been shown to mediate mPTP opening by acting as a final integrator of multiple signaling^27^. Moreover, we and others demonstrated that GSK3β inhibition was required for pre/postconditioning mediated resistance to ischemia-reperfusion (I/R) injury and mPTP opening^27–30^. Therefore, we decided to take advantage of in silico analysis of potential CypD phosphorylation sites for GSK3β to reveal a new CypD phosphorylation site crucial for mPTP sensitivity.

By combining genetic manipulations with both in vitro and in vivo mouse heart models, we demonstrated that the extent of myocardial damage is associated with the CypD phosphorylation on its S191 residue, thereby inducing its binding to OSCP, ROS production and the subsequent mPTP opening.

## Results

### CypD Phosphorylation at serine 191 regulates the mPTP opening

**Fig. 1:**
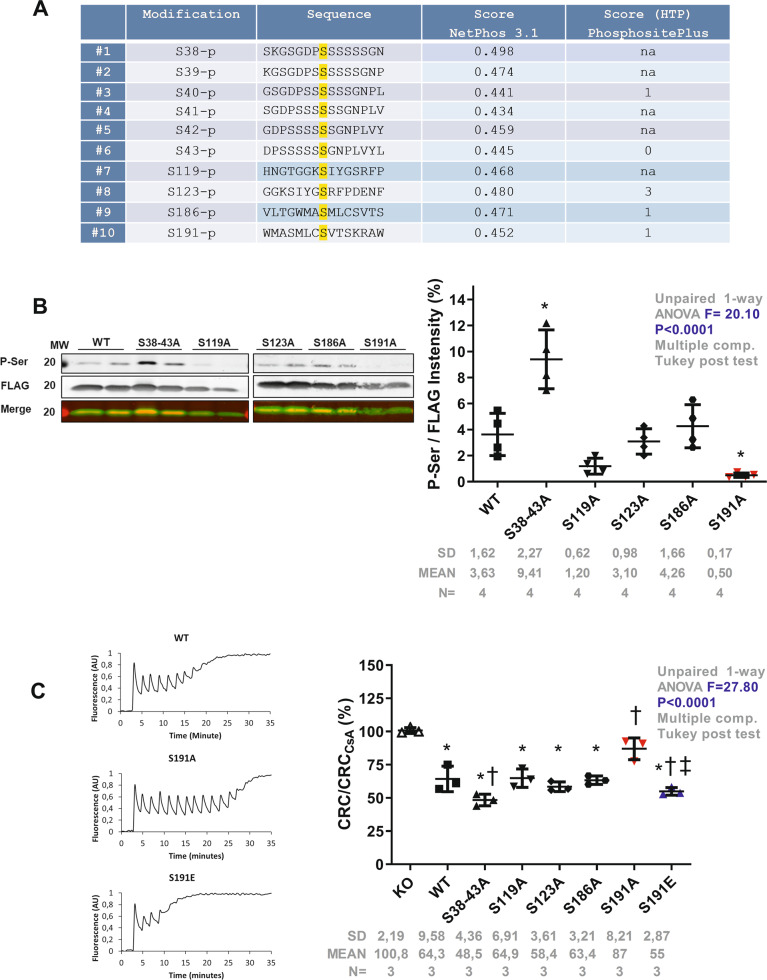
Multiple phosphorylation sites of CypD and mPTP regulation. **a** Table of the specific-GSK3β kinase post-translational phosphorylation site of CypD from the amino acid sequence Human PPIF (Uniprot #P30405)^50^. NetPhos 3.1 score of potential serine phosphorylation sites of CypD, and HTP (High Throughput papers) score from PhosphoSitePlus: number of records in which this modification site was assigned using only proteomic discovery mass spectrometry. Non applicable (na), phosphorylation (p). **b** Top: representative western blot of the mitochondrial fraction of HEK cells overexpressing FLAG-tagged mutants of CypD probed for phospho-serine (P-Ser) and FLAG. Merge was included to show that the P-Ser band is at the same level than the CypD-FLAG band. Bottom: Quantification of P-Ser/Flag intensities (mean ± SD, *n* = 4/group independent experiments) (*p < 0.05 vs. WT). Differences in means among multiple groups were analyzed using one-way ANOVA with a Tukey′s post hoc test. **c** Left: typical curves of calcium retention capacity (CRC) in HEK CypD-KO cells rescued with different CypD mutants. Right: Quantification of CRC in different CypD mutants normalized to the protein content and maximum protection afforded by 1 µM CsA. Differences in means among multiple groups were analyzed using one-way ANOVA with a Tukey′s post hoc test. (Mean ± SD, *n* = 3 independent experiments) (*p < 0.05 vs. KO, *†p* < 0.05 vs. WT, and *‡p* < 0.05 vs. S191A). CypD can be phosphorylated at multiple serine residues, but only the CypD phosphorylation at S191 seems to impact the ability of CypD to regulate the mPTP opening. Differences in means among multiple groups were analyzed using one-way ANOVA with a Bonferroni′s or Tukey′s post hoc test.

Post-translational modifications of CypD^26,31–33^ have been suggested to alter mPTP regulation. We and others have previously reported that inhibition of GSK3β abolishes mPTP opening^27,28,34,35^ and that GSK3β can phosphorylate CypD in vitro^26^. To investigate whether CypD may act as a potential substrate for GSK3β, we performed CypD-in silico analysis for putative GSK3β phosphorylation sites (S/T-x-x-x-S/T)^36^. In silico sequence analysis performed by NetPhos3.1 revealed 10 consensus phosphorylation sites on residues S38, S39, S40, S41, S42, S43, S119, S123, S186 and S191 (Fig. 1a), suggesting that CypD can potentially be phosphorylated at multiple serines by GSK3β with a predictive score averaging 0.462 ± 0.019 (Fig. 1a).

To evaluate their impact on CypD phosphorylation level, we individually mutated each identified serine to alanine. Since S38 to 43 are a series of serines close to the mitochondrial targeting sequence (MTS) cleavage zone and to avoid any neighboring serine phosphorylation, we have grouped them under a single mutant S38–43A. CypD-FLAG-tagged mutant proteins were then overexpressed in HEK cells. After FLAG immunoprecipitation, our results showed that mutants S119A, S123A and S186A did not significantly modify the global serine phosphorylation level of CypD, averaging, respectively, 1.20 ± 0.62%, 3.10 ± 0.98% and 4.26 ± 1.66% vs. 3.63 ± 1.62% in the WT group (*p* > 0.05 = ns, Fig. 1b). Surprisingly, the mutant S38–43A induced a significant increase of the serine phosphorylation level of CypD averaging 9.41 ± 2.27% (*p* = 0.0002), suggesting that serines S38 to S43 may act as a negative regulator of the phosphorylation status of CypD. More interestingly, when S191 was mutated to alanine, the CypD phosphorylation was significantly reduced, averaging 0.44 ± 0.09% vs. 3.63 ± 1.62% in the WT group (*p* = 0.0481) (Fig. 1b), suggesting a key role of the S191 in the global phosphorylation process of CypD.

We next tested whether identified mutations could alter mPTP opening. To this end, CRISPR/Cas9 was used to knock out endogenous CypD in HEK cells and subsequently rescued with either WT or mutated CypD (Supplementary Figure 2). As expected, WT cells exhibited a significant mPTP sensitization to the calcium load averaging 64.3 ± 9.6% vs. 100.8 ± 2.2% in the CypD KO group (*p* < 0.05) (Fig. 1c). In S119A, S123A and S186A mutants, the calcium retention capacity (CRC) was similar to the WT group, averaging 64.3 ± 6.9%, 58.4 ± 3.6% and 63.3 ± 3.2% respectively (*p* = ns vs. WT) (Fig. 1c). While the mutant S38–43A exhibited a significant reduction in CRC ratio, averaging 48.5 ± 4.4% (*p* < 0.05 vs. WT; Fig. 1c), the phosphoresistant mutant S191A was the only mutant to elicit significant protection as compared to WT group with a CRC ratio averaging 87.0 ± 8.2% and 64.3 ± 9.5%, respectively, (*p* < 0.05). These data suggest that throughout the putative serine residues identified in silico, only the phosphoresistant mutant S191A afforded a similar protection as loss of CypD in the regulation of the mPTP opening.

To reinforce the specificity of S191 in mPTP regulation, we designed a phosphomimetic mutant by mutation of serine 191 to glutamic acid (S191E). In these conditions, our results showed that the mutant S191E not only reversed the mPTP protection induced by the phosphoresistant S191A mutant (*p* < 0.05) (Fig. 1c), but also induced a significant sensitization of mPTP opening with a CRC ratio averaging 55.0 ± 2.9% vs. 64.3 ± 9.6% in the WT group (*p* < 0.05) (Fig. 1c). Altogether, these results suggest that the phosphorylation event of CypD at serine 191 controls the Ca^2+^-sensitive mPTP opening.

### CypD Phosphorylation at serine 191 regulates the binding of CypD to the mPTP core component

**Fig. 2:**
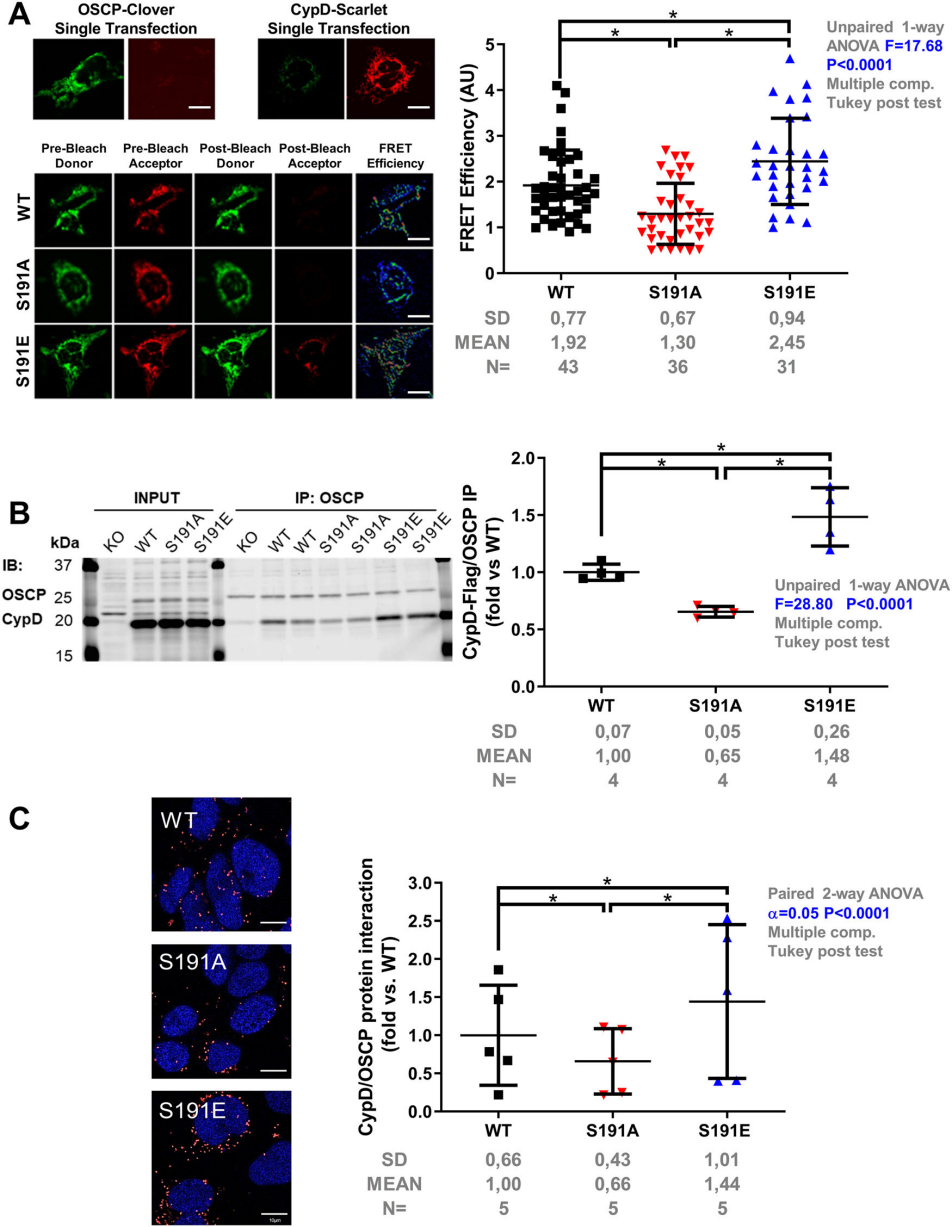
Phosphorylation event of CypD at serine 191 modulates the binding of CypD to the mPTP. **a** Left panel: Representative images of HEK cells transfected with OSCP-Clover with WT or CypD mutants Scarlet downstream of an IRES site. Scale bar 10 µm. Cells were excited with a 458 nm laser and emission at 505–540 nm and 575–620 nm with a 560 nm barrier filter. Right panel: Quantification of FRET efficiency in WT, phosphoresistant (S191A) and phosphomimetic (S191E) CypD mutants (mean ± SD; *n* = 31–43 on 3 different experimental days with ~10–12 cells per day) (*p < 0.05 vs. respective group). Differences in means among multiple groups were analyzed using one-way ANOVA with a Tukey′s post hoc test. **b** Left panel: Representative immunoblots of CypD and OSCP following OSCP immunoprecipitation in HEK CypD mutant cell lines. Right panel: Quantification of the immunoprecipitation OSCP probed for CypD in WT, S191A, and S191E CypD mutants (fold vs. WT mean ± SD; *n* = 4 independent experiments) (*p < 0.05 vs. respective group). Differences in means among multiple groups were analyzed using one-way ANOVA with a Tukey′s post hoc test. **c** Proximity ligation assay between OSCP and CypD in HEK CypD-KO cells rescued WT, S191A and S191E CypD mutants. Left panel: representative confocal microscopy images of in situ OSCP-CypD interactions depicted as red dots. Nuclei appear in blue. Scale bar 10 µm. Right panel: quantification of the interactions per cell presented as a fold of WT (*n* = 5 different experimental days with 4 fields of 20–50 HEK cells per well) (*p < 0.05 vs. respective group). Differences in means among multiple groups were analyzed using a two-way ANOVA followed by a Tukey′s post hoc test. Scale bar 10 µm. The phosphorylation event of CypD at serine 191 regulates the binding of CypD with the mPTP pore component OSCP.

It was recently demonstrated that CypD not only regulates the mPTP but also regulates the dynamic assembly of mitochondrial ATP synthase^37^. To further define the mechanism of how CypD regulates mPTP opening, we then investigated whether S191 residue plays a role in CypD binding with the mPTP. It has been recently reported that CypD could bind to the OSCP subunit of the ATP synthase, a proposed pore constituent^10,38^. We here hypothesized that CypD phosphorylation on its residue S191 induces its translocation and binding to the OSCP to favor mPTP opening. To test our hypothesis, we used three different strategies to measure the effect of CypD phosphorylation on the interaction of CypD with the pore. First, we designed a FRET system in which OSCP was tagged with Clover GFP (Fig. 2a). Downstream of an internal ribosomal entry site, either WT, phosphoresistant (S191A), or phosphomimetic (S191E) CypD was tagged with the FRET pair Scarlet (Fig. 2a). Our results showed that the FRET ratio between CypD and OSCP was significantly decreased in phosphoresistant S191A mutant averaging 1.29 ± 0.66 vs. 1.92 ± 0.76 in WT group (*p* < 0.05), whereas the phosphomimetic mutant S191E enhanced the FRET ratio up to 2.44 ± 0.94 (*p* < 0.05 vs. WT) (Fig. 2a). These results suggest that the OSCP/CypD interaction was under the influence of the phosphorylation event of CypD at S191. These results were confirmed by co-immunoprecipitation assays (Fig. 2b), showing that the protein interaction between OSCP and CypD was significantly reduced by 45% in the phosphoresistant S191A group, averaging 0.65 ± 0.04 vs. 1.00 ± 0.07 in WT group (Fig. 2b), whereas the phosphomimetic S191E mutant significantly increased the OSCP/CypD interaction by 48%, averaging 1.48 ± 0.25 as compared to the WT group (*p* < 0.05) (Fig. 2b). To further illustrate these results in situ, we finally used a PLA approach in our three mutant cell lines. As shown in Fig. 2c, the decreased interaction between OSCP with CypD afforded by the phosphoresistant S191A mutant was significantly reversed in the phosphomimetic S191E group (*p* < 0.05 vs. WT). These data suggest that the phosphorylation event of CypD at serine 191 regulates the binding of CypD to the OSCP subunit of the ATP synthase, which is known to sensitize mPTP opening^10,39^.

Interestingly, in basal condition, although oxidative phosphorylation did not seem to be different in WT, S191A, and S191E mutants (Supplementary Fig. 3A), the mortality and the ROS production of cells were significantly higher in the S191E phosphomimetic mutant as compared to the WT group (*p* < 0.05, Supplementary Fig. 3B, C), suggesting that the enhancement of the CypD/OSCP interaction may predispose cells to injury and death.

### CypD Phosphorylation at serine 191 regulates cell death and infarct size

**Fig. 3:**
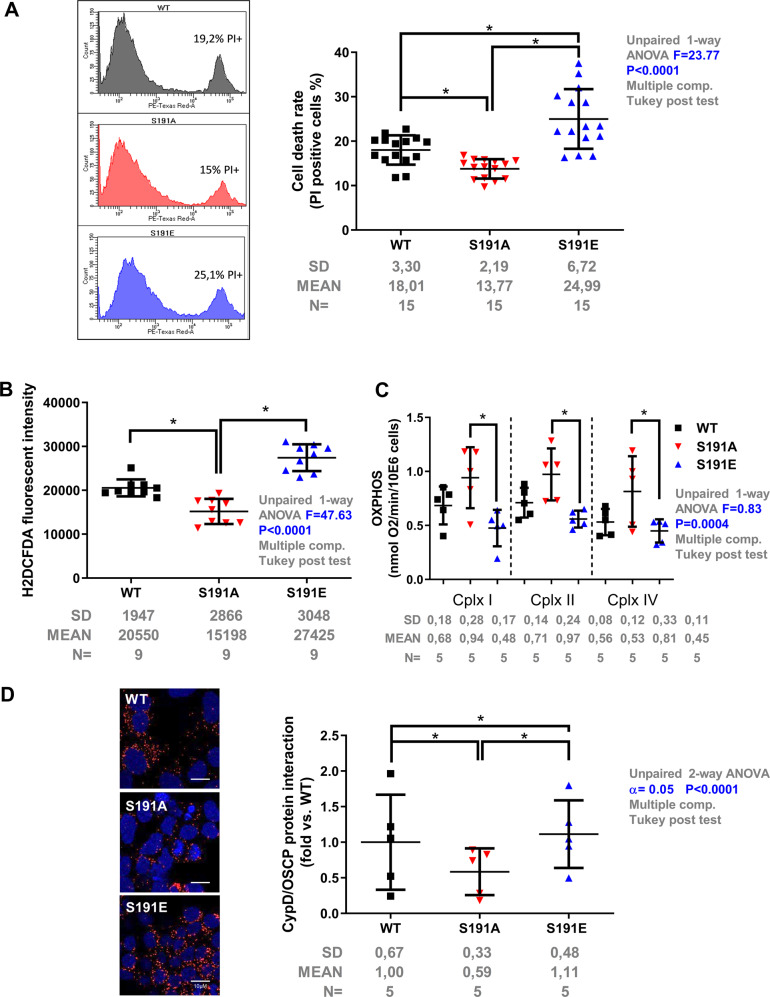
Phosphorylation of CypD at serine 191 regulates mitochondrial function and cell death. **a** Left panel: typical cytometry histograms of PI staining in different CypD mutant cell lines, with the corresponding percentage of PI positive cells. Right panel: Percentage of cell death measured by flow cytometry after 18 h starvation stress in WT, phosphoresistant (S191A) and phosphomimetic (S191E) CypD cell lines. Differences in means among multiple groups were analyzed using one-way ANOVA with a Tukey′s post hoc test. (mean of PI-positive cells ± SD, *n* = 15 different experimental days with 10 000 events/assay) (*p < 0.05). **b** ROS level production of mutants measured by flow cytometry (mean of H2DCFDA fluorescent intensity ± SD, *n* = 9 different experimental days with 10 000 events/assay) (*p < 0.05). Differences in means among multiple groups were analyzed using one-way ANOVA with a Tukey′s post hoc test. **c** Oxidative phosphorylation measured on permeabilized CypD mutant cell lines after 18 h starvation stress. After permeabilization of CypD mutant cells with digitonin (10 μg/ml), consumption of oxygen was strongly activated by 2 mM ADP. Successive additions of rotenone (0.5 μM), succinate (complex II substrate; 10 mM), TTFA (40 μM), TMPD/ascorbate (complex IV substrates; 0.3 and 3 mM respectively), and then azide (15 mM), allowed determination of sensitive rates of oxidative phosphorylation using complex I, II, and IV substrates, respectively (mean of sensitive respiration rate ± SD, *n* = 5 different experimental days with 1–2 million cells/assay, *p < 0.05). Differences in means among multiple groups were analyzed using one-way ANOVA with a Tukey′s post hoc test. **d** Proximity ligation assay between OSCP and CypD in HEK CypD-KO cells rescued with WT, S191A or S191E CypD mutants following 18 h starvation. Left panel: representative confocal microscopy images of in situ OSCP-CypD interactions depicted as red dots. Nuclei appear in blue. Scale bar 10 µm. Right panel: quantification of the protein-protein interaction presented as fold of WT (*n* = 5 different experimental days with 4 fields of 20–50 HEK cells per well) (*p < 0.05). Differences in means among multiple groups were analyzed using a two-way ANOVA followed by a Tukey′s post hoc test.

Since mPTP has long been known as one of the main regulators of cell death^40^; and it has been suggested that OSCP can modulate ATP synthase function and mediates mPTP opening^9,10^, we next sought to determine whether the phosphorylation of CypD at S191 affects cell death following serum-nutrient starvation, which is a stress model known to induce cell death through mitochondrial depolarization and mPTP opening^41,42^. Cell death was significantly increased in the S191E mutant in comparison to S191A after 18 h starvation with a relative cell death averaging 24.99 ± 6.72% and 13.77 ± 2.18%, respectively, (Fig. 3a). In these conditions, the increase of the ROS production measured by flow cytometry in S191E mutant was significantly abrogated in S191A group (*p* < 0.05) (Fig. 3b). Moreover, measuring mitochondrial respiration after starvation with complex I, II or IV substrates, our results showed that oxidative phosphorylation was reduced by half in the S191E group as compared to the S191A cells (*p* < 0.05) (Fig. 3c). Altogether, these results suggest that the phosphorylation event of CypD on its S191 residue controls cell death by alteration of mitochondrial function.

It is worth noticing that the protein interaction of CypD with OSCP was positively correlated with the level of cell death. Indeed, as shown in the Fig. 3d, while the protection provided by the S191A mutant displayed significantly lower interactions of CypD with OSCP (*p* < 0.05 vs. S191E), these interactions were significantly increased in injured S191E mutant cells after starvation. Altogether, these data demonstrate that CypD phosphorylation at S191 enhances binding of CypD to the OSCP, alteration of mitochondrial function, and subsequent cell death, suggesting that residue S191 is important in this regulation.

Lastly, we tested whether the phosphorylation level of CypD regulates in vivo I/R injury using a mouse model. To rule out a possible effect of the adenovirus deliverance and/or infection process on the infarct size by itself, a pilot study using GFP adenovirus was used as a positive infection control. As shown in the Supplementary Fig. 4A, B, at 1 week after in vivo gene delivery, the overexpression of GFP in the ventricle covered 35.2 ± 12.1% of the area at risk (AR). In these conditions, with a comparable AR (*p* = ns, Supplementary Fig. 4B) and without any inflammation sites (data not shown), the GFP overexpression did not modify the infarct size compared to the CTRL group (*p* = ns, Supplementary Fig. 4B, C). These results demonstrate that our virus injection protocol by itself has no effect on the infarct size, suggesting that any observed infarct modulation after a gene delivery is due to the direct gene expression rather than to the adenoviral delivery process.

**Fig. 4:**
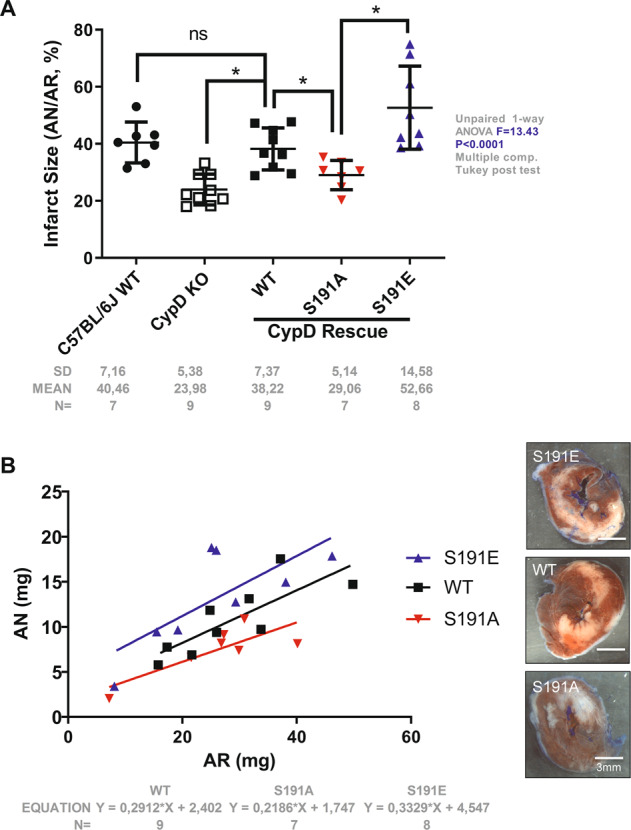
Phosphorylation event of CypD at S191 regulates Myocardial infarction. **a** Infarct size (AN) expressed as a % of the area at risk (AR) in C57BL/6J, CypD KO and CypD mutant rescue (WT, S191A and S191E) mice (mean ± SD, *p < 0.05 vs. respective group). Differences in means among multiple groups were analyzed using one-way ANOVA with a Tukey′s post hoc test. **b** Scatterplot of AN over the AR of CypD KO hearts infected with 5 × 10^8^ WT, S191A, or S191E CypD virus particles per mouse. Area of necrosis was positively correlated to the AR in WT, S191A and S191E groups with a Pearson *r* value of 0.806, 0.802, and 0.747, respectively. Most data points for S191A group were below the WT rescue regression line, indicating that for any size of AR, these hearts developed significantly smaller infarcts. Conversely, most data points for S191E rescue mice were above the WT regression line, indicating that these hearts developed higher infarcts, suggesting that the phosphorylation event of CypD at S191 controls the extent of infarct size at reperfusion. Scale bar 3 mm.

Therefore, we generated three adenovirus constructs containing either the WT, the phosphoresistant S191A, or the phosphomimetic S191E CypD mutant which were injected into the anterolateral wall of the left ventricle of CypD-KO mice. After 7 days of recovery, CypD-rescued mice underwent 45 min ischemia followed by 24 h reperfusion. The AR was comparable among groups, ranging from 25.5 ± 10.5% to 28.8 ± 11.2% of the LV (*p* = ns among groups) and the CypD protein rescue level was also comparable among groups (*p* = ns) (Supplementary Fig. 5A), with a specific localization into mitochondria (Supplementary Fig. 5B). Mice rescued with the WT CypD exhibited a significant increase of infarct size compared to CypD KO mice, averaging 38.2 ± 7.4% and 23.9 ± 5.3% of AR respectively (*p* < 0.05) (Fig. 4a). It is worth mentioning that the infarct size of these CypD WT-rescued mice was equivalent to our standard C57BL/J6 mice (*p* = ns; Fig. 4a). When necrosis was plotted with area at risk (Fig. 4b), most data points for S191A group were below the WT rescue regression line, indicating that for any size of AR, these hearts developed significantly smaller infarcts, averaging 29.0 ± 5.1% vs. 38.2 ± 7.3% of AR in WT (*p* < 0.05 vs. WT) (Fig. 4a, b). Conversely, most data points for S191E rescue mice were above the WT regression line, indicating that these hearts developed not only higher infarcts, averaging 52.6 ± 14.5% of AR (*p* < 0.05 vs. WT) (Fig. 4a, b), but also higher mortality at reperfusion (Supplementary Table 2). In summary, these data suggest that the phosphorylation event of CypD at S191 induces the binding of CypD to OSCP, favoring mPTP opening and subsequent cell death at reperfusion.

## Discussion

The present study suggests that the CypD phosphorylation at S191 controls cell death and the extent of myocardial infarction in vivo, via the modulation of the mPTP opening which is dependent of the binding state of CypD with OSCP, the mitochondrial electron transport chain coupling and the ROS generation.

Although the pore structure is still debated, the mechanism through which CypD regulates mPTP opening is the subject of intensive studies. Post-translational modifications of CypD have been shown to modulate pore opening^22,26,31–33^. Based on publications suggesting that GSK3β/CypD complex plays a pivotal role in the regulation of the mPTP^26,28,35,43^, and although it has been reported that the consensus sequence S/T-X-X-X-S/T is neither essential nor sufficient to guide GSK3β-dependent phosphorylation^39,44^, our in silico analysis shows that CypD presents no less than ten potential target sites for GSK3β. Even if the role of GSK3β on CypD has been recently questioned^45^, these results are in line with a previous report showing that GSK3β can directly phosphorylate CypD at Ser/Thr in vitro^26^. We assume that these preferential domain-specific kinase-substrate relationships can be used only to distinguish cognate kinase-substrate pairs from all other non-cognate combinations and that further studies are required to provide the proof that CypD is a bona fide substrate for GSK3β.

In this context, we show that the global phosphorylation level of CypD is dependent on the phosphorylation of some in silico identified serines and that the phosphorylation event of CypD seems to control the mPTP opening. Our results are in line with recent reports showing that the phosphorylation of CypD could be associated with increased mPTP opening^22,26^. The study by Parks et al. addressed the question why the hearts from global MCU-KO mice are not protected from ischemic injury by investigating whether adaptive alterations occur in cell death signaling pathways. Their results show that MCU-KO mitochondria exhibit an increase in phosphorylation of CypD-S42 which decreases mPTP calcium sensitivity thus allowing activation of mPTP in the absence of an MCU-mediated increase in matrix calcium. Using a phosphoresistant S42A strategy, they conclude that the phosphorylation of CypD at S42 seems to regulate mPTP. However, in the present study, in silico results show that CypD can be also potentially phosphorylable on neighboring serines 38, 39, 40, 41, and 43. Since making a targeted mutation into a residue-long flanking regions is known to involve a risk for the kinase to phosphorylate neighboring serines +1, 2, 3 or 4^46^, their mutant S42A has an high risk to have a phosphorylation compensation which was not discussed. For this reason, we chose to regroup all serines in a single mutant S38–43A, avoiding any possible phosphorylation compensation in neighboring serines. Our results show that the S38–43A mutant significantly increases the global phosphorylation level of CypD associated with a significant decrease of the CRC as compared to the WT group. Conversely to Parks′ results, these results might suggest that the multiple mutations of serines could potentiate the phosphorylation of CypD and the over-sensitization of the mPTP opening. Altogether, these results suggest that the domain S38–43, located just after the MTS and before the cyclophilin-isomerase-domain, seems to play a role in the phosphorylation process of CypD and the activation of the mPTP opening. But the mechanism by which this domain regulates mPTP opening by phosphorylation, modification of the protein addressing and/or the modification of the isomerase activity requires additional work.

On the other hand, our results show that only the phosphoresistant mutant S191A is able to completely abolish the phosphorylation of CypD, and confers resistance of mPTP opening against calcium. Since cardioprotection requires inhibition of multiples kinases^28,47^, these results suggest that even if other serines are functionally actives (including S42), S191 seems to plays a preponderant role on the regulation of mPTP opening through the phosphorylation event of CypD^26^.

Growing data indicate that CypD, in addition to its role in mPTP, may play a pivotal role in regulating overall cell metabolism. Studies including genetic or pharmacological inhibition of CypD revealed its contribution to the regulation of respiratory function^48,49^ and oxidative phosphorylation^49,50^. Our results add to this by showing that the phosphorylation event of CypD at S191 regulates mitochondrial function, such as OXPHOS and ROS production. Indeed, while protected phosphoresistant S191A cells exhibit improved mitochondrial respiration associated with a reduction of ROS production, phosphomimetic S191E cells are more sensitive to cell death stress due to an alteration of oxidative phosphorylation and consecutive ROS production. Thus, our result are in line with previous studies showing that the Ca^2+^ overload induced by the extensive ROS generation causes necrosis through the enhancement of the permeability of the mitochondrial membrane and mPTP opening^51,52^. Altogether, our results suggest that CypD phosphorylation at S191 not only regulates mPTP opening but also seems to be a crucial regulator of ROS production, underlining the key role of the triangle CypD-phosphorylation/ROS-production/mPTP-opening in cell death pathway, especially during ischemia-reperfusion in the heart in vivo^51,52^.

In this context, the most significant finding to emerge from this study is that the extent of myocardial damage is dependent on the CypD phosphorylation event on its S191 residue. To our knowledge, this is the first in vivo research study to have demonstrated that the phosphorylation of CypD on a precise serine residue is involved in acute myocardial infarction. Our results show not only that the infarct size is dependent of the CypD-S191 phosphorylation, but also that phosphorylation inhibition of this same serine significantly reduces the infarct size, providing new insight in cardioprotection. Moreover, although we did not observe any heart rate alteration or any arrhythmic event after the CypD rescue in our acute mouse model (data not shown), these potential mechanisms underlying S191 phosphorylation of CypD are of interest for future LV remodeling studies.

Regarding possible mechanisms for the modulation of mitochondrial function and subsequent cell death in our model, in addition to post-translational modifications, it has been demonstrated that interaction with proteins in the matrix and IMM can modulate the CypD activity and mPTP opening. Numerous studies provided evidence that CypD can interact with other proteins including ANT^53,54^, P_i_C^55^ and the F_O_F_1_-ATP synthase subunit OSCP^50^ and thus stimulate mPTP induction. Our data further show that the CypD/OSCP interaction is positively correlated with the sensitization of mPTP opening and that the phosphorylation event of CypD at S191 regulates the binding of the CypD with the mPTP.

One may question whether mitochondrial calcium entry may be affected in our mutants (i.e. MCUR1)^56^. Such mechanisms remain to be investigated in-depth in future studies. However, although we notice a difference of CRC in our mutants, the slope of mitochondrial calcium uptake is similar in all groups (Fig. 1c) suggesting that our mutants exhibit more a difference in the threshold of mPTP opening rather than a calcium entry dysfunction.

**Fig. 5:**
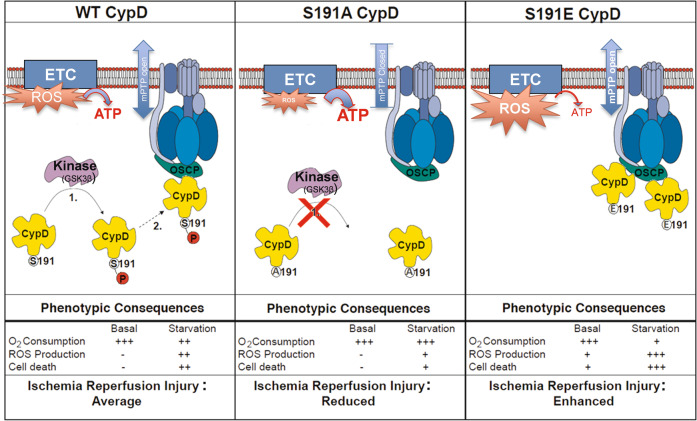
Proposed GSK3β/CypD signaling axis for mPTP regulation. Under baseline condition (WT group), the phosphorylation of CypD by a kinase (e.g. GSK3β) at S191 induces its translocation to the OSCP, to favor mPTP opening and subsequent cell death at reperfusion. When CypD cannot be phosphorylated in S191A mutant, the CypD/OSCP interaction is reduced, maintaining mPTP in its closed conformation and providing protection against cell death. In contrast, in phosphomimetic S191E mutant cells, the interaction of CypD with the OSCP is amplified, which is associated to a mitochondrial dysfunction to enhance ischemia-reperfusion injury.

In summary, our work identifies S191 of CypD as a key regulator of ROS production, mPTP opening and cell death. As illustrated in Fig. 5, we propose that CypD phosphorylation at S191 induces the translocation and favors binding of CypD to OSCP, which is associated to an OXPHOS alteration, increased ROS production, and mPTP opening leading to subsequent cell death at reperfusion.

## Material and methods

Material and methods are detailed in the Supplemental methods and figure legends. The data, analytic methods, and study materials will be/have been made available to other researchers for purposes of reproducing the results or replicating the procedure.

### Animals

Experiments were conducted in accordance with the Guide for the Care and Use of Laboratory Animals (NIH Publication No. 85–23, revised 1996), and were approved by local institutional animal research committees′ #19896–201903212127912 and #901I.

### Starvation protocol

HEK cells were serum starved in serum-free high-glucose DMEM in the presence of pyruvate for 18 h^57^. Following starvation, cells were collected and resuspended in PBS or respiration buffer before analysis.

### In vivo model of ischemia/reperfusion (I/R)

Mice were randomized to receive 25 µl of an adenovirus solution (CypD mutants at 5 × 10^8^ PFU), which was injected in 10–12 different sites of the LV wall to cover the maximum area of the area at risk^58^. The chest cavity was closed, and the mice were allowed to recover for 1 week prior to I/R surgery. Mice underwent I/R surgery as previously described^29^ (Supplementary Fig. 1).

### Statistical analysis

Data for multiple experiments were quantified and expressed as mean ± SD where indicated. Differences in means among multiple groups were analyzed using one-way ANOVA with a Bonferroni’s or Tukey’s post hoc test to determine significance between groups. For results with two groups, normal distribution of values was verified using the Kolmogorov–Smirnov test and the differences between groups were then determined using a two-tailed paired students *t*-test. For results with 2 variables, a two-way ANOVA followed by a Tukey′s post hoc test was performed. Statistical significance was set at a threshold of *p* ≤ 0.05. No data/animals were excluded from the study. The data were computed using GraphPad Prism 6.1 software.

## Supplementary information

Supplmental methods and figure legends

Supplemental Tables

Supplemental Figure 1

Supplemental Figure 2

Supplemental Figure 3

Supplemental Figure 4

Supplemental Figure 5
