# Supplementary material for: Phosphorylation of cyclophilin D at serine 191 regulates mitochondrial permeability transition pore opening and cell death after ischemia-reperfusion
Source: Cell Death Dis. 2020 Aug 19;11(8):661. doi: 10.1038/s41419-020-02864-5 (PMC7438327; doi:10.1038/s41419-020-02864-5)
Supplement: Supplementary file 1 — Supplmental methods and figure legends [file 41419_2020_2864_MOESM1_ESM.pdf]

Supplemental methods and Figure legends

Material and Methods

Animals

All experiments were conducted in accordance with the *Guide for the Care and Use of Laboratory Animals* published by the US National Institute of Health (NIH Publication No. 85-23, revised 1996), and were approved by local institutional animal research committees' #19896-201903212127912 and #901I. All experiments were performed on male mice of 8-12 weeks of age. CypD-KO mice on a C57BL/6 genetic background were a gift from S. J. Korsmeyer's laboratory (Boston, MA, USA)[1] and C57BL/6J mice were from Charles River Laboratory.

Cell culture

Human embryonic kidney (HEK) 293T (ATCC, CRL-3216) were grown in high-glucose DMEM (Fisher Scientific) supplemented with 10% heat-inactivated fetal bovine serum (Gibco) and 1% penicillin/streptomycin (Corning). Cells were kept in an incubator at 37°C with 5% CO_2. Cells were tested for mycoplasma contamination with the MycoAlert kit (Lonza, LT07-418).

Plasmid generation

Vector and insert were annealed and transformed using ligation independent cloning as previously described[2]. Plasmids were verified by DNA sequencing (Genscript). Cyclophilin D mutants were generated using Q5-site-directed mutagenesis and the primers in Supplemental Table 1.

Development of CRISPR knockout cell lines

The Cas9 D10A NIckase-2A Puro enzyme and U6 promoter and empty sgRNA site (Addgene) were each subcloned into modified pRSET vector with either a CMV LIC2 BGH promoter system or with no promoter and terminator sequence. The sgRNAs were inserted into the empty sgRNA guide vector via Q5-site-directed mutagenesis and verified by DNA sequencing (Genscript) using the U6 Fwd Seq primer. Each sgRNA with promoter was PCR amplified and purified for Golden Gate Assembly to form a functional pair of guide RNAs using the primers indicated in Supplemental Table 1. The backbone for the guide RNA was from pEGFP-C1 with a Bsa1 site mutated via site-directed mutagenesis. The Golden Gate reaction was performed with 60 ng PCR amplified backbone, 30 ng of each guide RNA, 10000 units Bsa1, 10000 units dpnI, 200,000 units T4 Ligase, and 2 µL of 10x T4 Buffer (New England Biolabs) brought to a final volume of 20 µL. The Golden Gate Assembly reaction was incubated in a thermocycler under the following conditions: 37°C for 5 minutes, 15°C for 15 minutes, 37°C for 60 minutes, and 55°C for 5 minutes. 5 µL of the assembly reaction was transformed into NEB 10-beta bacteria. Final sgRNA vector was verified by DNA sequencing (Genscript) using the sgRNA seq Fwd and sgRNA seq Rev primers.

HEK 293T cells were transfected with 1 µg Cas9D10A nickase plasmid and 2 µg of the sgRNA pair plasmid. Cells were selected with 1 µg/mL of puromycin and 1600 µg/mL G418. Single cells were then clonally isolated, and knockout was verified by Western blot.

Western blotting

Samples were diluted in Laemmli’s buffer (60 mM tris, 5% β-mercaptoethanol, 2% sodium dodecyl sulfate, 10% glycerol, 0.01% bromophenol blue) and loaded onto a

13.5% SDS-PAGE gel submerged in running buffer (25 mM tris, 192 mM glycine, 0.01% SDS). Samples were initially run at 80V for 15 minutes to reach a resolving gel and then run at 200 V for 50 minutes in running buffer. The sample was then transferred to nitrocellulose membrane (BioRad #1620115) in a tank containing transfer buffer (25 mM tris, 192 mM glycine, 20% methanol) and run either at 30 V 4°C overnight or 100 V 90 minutes on ice. The membrane was washed and stained with ponceau red solution (0.4% w/v ponceau 5% w/v acetic acid) and checked for proper transfer. The membrane was then washed with ddH_2O and blocked with Odyssey Blocking Buffer (LI-COR, 927-40003). The membrane was then probed with primary antibody diluted in PBS-T (0.05% Tween) for 2 hours at room temperature or 4°C overnight, (1:1000) except for: PPIF (Abcam 1:5000), FLAG (Genscript 1:2000), Flag (Cell Signaling Technologies 1:2000), OSCP (Santa Cruz Biotechnology 1:200), PhosphoSerine (Millipore 1:200), phospho-serine (Qiagen 1:200), and GAPDH (Cell Signaling Technologies 1:5000). Membrane was then washed 3 times with PBS-T (0.01% Tween) and probed with secondary antibody (1:20,000, LI-COR). The membrane was observed on an Odyssey Classic machine (LI-COR). The image was analyzed using Image Studio Lite software (LI-COR).

Calcium retention capacity (CRC)

HEK 293T cells were counted and resuspended to a concentration of 12.5×10^6 cells/mL in mitochondrial assay buffer (20 mM tris, 150 mM sucrose, 50 mM KCl, 2 mM KH_2PO_4, and 5 mM succinate, 20 µM EGTA). 300 µL of cell suspension (3.75×10^6 cells) was transferred to each of 4 cuvettes containing 1.7 mL mitochondrial assay buffer supplemented with 2 µM thapsigargin and 0.5 µM Fura2-FF. 1 µM of CsA was added to 2 of the 4 cuvettes. Cuvettes were placed into a Cary Eclipse fluorescent

spectrophotometer. Digitonin (20 µM) was added to at 1 minute and, starting at 5 minutes, 40 nmol of $CaCl_2$ was added and 10 nmol of $CaCl_2$ every 2 minutes after until mPTP was opened. The quantification of CRC in different CypD mutants was first normalized by the protein content and further to the maximum protection afforded by 1 µM CsA.

Starvation protocol

HEK293T cells were grown in polystyrene six-well plates with DMEM supplemented with 4 mM L-glutamine (Gibco, 41966-029), 10% fetal calf serum (Gibco, 10270-106), 1 mM sodium pyruvate (Gibco, 11360-039), 100 µM nonessential amino acids (Gibco, 11140-035) and 100U/mL penicillin, 100 µg/mL streptomycin (Gibco, 15140-122) at a concentration of 280 000 cells/well. Twenty four hours later, serum-containing medium was removed, and cells were carefully washed twice with warm sterile PBS (Gibco/Invitrogen 10010-015). HEK cells were serum starved in serum-free high-glucose DMEM in the presence of pyruvate for 18 hours[3]. Following starvation, collected cells after 5 minutes accutase incubation at 37°C were pooled to respective supernatant. After centrifugation 5 minutes at 500 g, supernatants were discarded and pellets were resuspended in PBS or respiration buffer before analysis.

Mitochondria isolation

Mitochondria were isolated from cultured cells in mitochondrial assay buffer. Cells were then lysed by passing them through a 10 mL syringe with a 27.5 gauge needle (BD # 305109) 6 times. For isolation from tissue, samples were crudely processed with scissors and then transferred to a chilled homogenizer tube with 1 mL of mitochondria assay buffer with protease and phosphatase inhibitor cocktails (Biotool). Cells were

homogenized at 160 RPM using a Dounce homogenizer. Mitochondria were separated through differential centrifugation; the resulting pellet was lysed in 1x lysis buffer (Cell Signaling Technologies) with protease and phosphatase inhibitor cocktails (Biotool).

FRET analysis

HEK 293T cells transfected with OSCP-Clover IRES CypD-Scarlet or mutant S191A/S191E CypD Scarlet were plated on 35 mm glass-bottom dishes (Matek). Cells were observed using an FV1000 confocal microscope (Olympus) and excited using a 458 nm laser and emission at both 505-540 nm and 575-620 nm with a 560 nm barrier between them. Images were analyzed using ImageJ software. Corrected total cell fluorescence for each channel was calculated for each cell imaged. The corrected fluorescence intensity of the Scarlet was then divided by the corrected intensity of the Clover emission.

The experiments were repeated three times with ~10-12 cells per day and the average value of fluorescence were taken at each specific condition.

Immunoprecipitation

400 µg of protein lysate was incubated with 1 µg of the indicated antibody overnight at 4°C on a rotator. 30 µL of SureBeads A&G Mixed 1:1 (BioRad, 1614833) were added and incubated on a rotator at 4°C for 2 hours. Samples were placed near a magnetic rack, and the supernatant was aspirated off. Samples were washed 2 times with 500 µL PBS-T (0.01% Tween). Beads were incubated with 2x Laemmli buffer and boiled for 10 minutes. Samples were placed near a magnetic rack and supernatant was transferred to a new tube ready to be analyzed by Western blotting.

Proximity Ligation Assay (PLA)

Millicell EZ slide 8-well glass plates (PEZGS0816) were coated with poly-D-lysine hydrobromide (Sigma P6407) to facilitate HEK binding. HEK 293T cell lines were plated (40K cells/well) for 8 hours, then wells were divided equally into control (basal) and under starvation conditions. After 18 hours of starvation, cells were fixed with 4% paraformaldehyde for 10 minutes at RT, then permeabilized with 0.01% Triton X-100 for 15 mins. Subsequently, PLA was performed according to the manufacturer's protocol (Sigma) with duolink kits anti-Goat plus (DUO92003) and anti-Mouse minus (DUO92004) and as previously described[4]. Primary antibodies used here were PPIF (1/200, sc82571) and OSCP (1/200, sc365162). Image acquisition was done using laser scanning confocal microscope (Nikon A1R, laser wavelengths 400-405 nm for DAPI and 561 nm for red dots) with a 60x oil-immersed objective.

The quantification of blobs per nucleus was blindly done with the BlobFinder software (Centre for Image Analysis, Uppsala University) and expressed as a fold of WT.

Oxidative phosphorylation (OXPHOS)

HEK cells (~$1.2x10^6$ cells) were suspended in respiration buffer containing 100 mM KCl, 1 mM EGTA, 5 mM KH_2PO_4 and 1 mg/ml BSA in 50 mM Tris/HCl (pH 7.4). A multiple substrate-uncoupler-inhibitor titration protocol was used with a high-resolution oxygraph (Oxygraph-2k; Oroboros, Innsbruck, Austria) to determine oxidative phosphorylation. Pyruvate/Malate/Glutamate (5 mM each) were used as complex I substrates. After permeabilization of HEK cells with digitonin (10 µg/ml), consumption of oxygen was strongly activated by 2 mM ADP. Successive additions of rotenone (0.5 µM), succinate (10 mM), TTFA (40 µM), TMPD/ascorbate (0.3 / 3 mM),

and then azide (15 mM) allowed determination of inhibitor sensitive rates of complex I, II, and IV respectively. Oxygen consumption was evaluated by the Oroboros DatLab4 software and expressed as nanomoles of oxygen per minute per million of cells. The final inhibitor sensitive *rate of each complex* was obtained by subtracting the corresponding *inhibitor-sensitive* respiration.

Flow cytometry

ROS detection was realized by using H2DCFDA (FP-467312, Interchim). Cells were incubated 30 minutes at 37°C with H2DCFDA at 1 µM. Following incubation, cells were washed with PBS. After centrifugation 5 minutes at 500 g, supernatants were discarded and pellets resuspended in PBS before flow cytometry analysis. Propidium iodide (PI, P4864 Sigma Aldrich), a cell viability probe, was added extemporaneously before acquisition at 1 µg/mL. Flow cytometry experiments were conducted blindly using Fortessa X-20 (BD Biosciences). 10,000 events by tube were acquired. Data were analyzed by DIVA Software (BD Biosciences). PI and H2DCFDA were excited respectively at 561 nm and 488 nm. The band pass filters collected the fluorescence of PI (620/20 nm) and H2DCFDA (530/30 nm). PI Cell death was represented by the percentage of positive cells for PI staining whereas the ROS production was expressed by relative fluorescence intensity.

In vivo model of ischemia/reperfusion (I/R)

A pre-specified effect size was previously calculated with Gpower 3.1 with α=0.05 and β=0.8 to determine size of different groups. 8-12 weeks old male CypD-KO mice underwent first CypD rescue procedure and then I/R surgery as previously described [5] (Chronogram of surgery protocol in Suppl. Figure 1).

Under anesthesia, the chest cavity was opened and mice were randomized to receive 25 µl of an adenovirus solution (CypD mutants at $5x10^8$ PFU), which was injected in 10-12 different sites of the left ventricular wall to cover the maximum area of the area at risk[6]. The chest cavity was closed, and the mice were allowed to recover for 1 week prior to I/R surgery.

Mice underwent 45 minutes of ischemia, followed by 24 hours reperfusion. Then, the area at risk was assessed by Evans Blue injection, and the area of necrosis was determined by TTC staining. Infarct size was blindly quantified with SigmaScan pro 5 software. No animals were excluded and report of death was resumed in the suppl. Table2.

Suppl. Figure 1: Chronogram of mice surgery

At Day 0, Mice underwent adenovirus injection (10-12 injection sites) in the potential area at risk. After having closed the chest cavity, mice were back to the animal facility for 7 days. At day 7, chest was re-open to do induce 45 minutes ischemia of LAD followed by 24 hours reperfusion. Illustration realized thanks to *Servier* Medical Art (*https://smart.servier.com/*).

<u>Suppl. Figure 2: CypD (Flag) rescue in HEK CypD mutant cells</u>

Top: Typical western blot of different CypD mutant cell lines. Membrane was probed for both CypD and FLAG with GAPDH as loading control. Bottom: Merge of tagged CypD with FLAG. Adenovirus constructs were generated using invitrogen's ViraPower Adenovirus system. The constructs encode for CypD-Flag IRES nuclear BFP driven by a CMV promoter. The larger size CypD was 3 tandem Flag Tags (GRSAEKLATMDYKDDDDKGSGDYKDDDDKGSGDYKDDDDKGS*) whereas the smaller ones (WT & S191 Mutants) were tagged with Sigmas' 3xFlag tag(GRSAEKLDYKDHDGDYKDHDIDYKDDDDKGS*) for improved co-IP immunogenicity.

<u>Suppl. Figure 3: OXPHOS, Cell death and ROS production in basal CypD-mutants :</u>

(A) After permeabilization of CypD mutant cells with digitonin (10 µg/ml), consumption of oxygen was strongly activated by 2 mM ADP. Successive additions of rotenone (0.5 µM), succinate (complex II substrate; 10 mM), TTFA (40 µM), TMPD/ascorbate (complex IV substrates; 0.3/3 mM), and then azide (15 mM), allowed determination of sensitive rates of oxidative phosphorylation using complex I, II, and IV substrates,

respectively. (Mean of sensitives respiration rate ± SD, n=6 different experimental days with 1.8 million cells/assay. (B) Basal mortality measured by flow cytometry in WT, phosphoresistant (S191A) and phosphomimetic (S191E) CypD cell lines. (Mean of PI-positive cells ± SD, n=12 different experimental days with 10 000 events/assay) (*p<0.05 vs. respective group). (C) Basal ROS production measured by flow cytometry (mean of H2DCFDA fluorescent intensity ± SD, n=3 different experimental days with 10 000 events/assay) in HEK CypD mutants (*p<0.05 vs. respective group). Differences in means among multiple groups were analyzed using one-way ANOVA with a Bonferroni's or Tukey's post hoc test.

Suppl. Figure 4: Effect of Adenovirus infection (GFP) on infarct size :

(A) Typical images of the virus injection after 7 days recovery and ischemia-reperfusion. Left: infected area at risk after blue staining. Middle: Color change of infected area at risk to emphasis color contrast (Adobe Photoshop7.0). Right: infected area of necrosis after TTC staining. (B) Area at risk expressed as % of LV (AR/LV), Infarct size expressed as a % of the area at risk (AN/AR) and area of positive virus infection (GFP+/AR) in CTRL and GFP infected mice (mean ± SD, *p<0.05 vs. respective group. n=5-7/gr). The differences between groups were then determined using a two-tailed paired students t-test.

(C) Scatterplot of AN over the AR of mouse hearts infected with $5x10^8$ GFP virus particles per mouse or veh. (CTRL). n=5-7/gr. Our results demonstrate that a "therapeutic dose" of virus injection protocol by itself has no effect on the area at risk and the infarct size, suggesting that any observed infarct modulation after a gene delivery is due to the direct gene expression rather than to the adenoviral process.

Suppl. Figure 5: Level of CypD-rescue into AR of CypD-KO mice

A. Top. Western blot of CypD from area at risk (collected after TTC staining) of C57BL/6J, CypD-KO, and CypD-WT (WT), CypD-S191A (S191A), CypD-S191E (S191E) rescued mice. Relative intensity in percentage of CypD versus C57BL/6J WT (mean ± SD, n = 5-7/gr). Differences in means among multiple groups were analyzed using one-way ANOVA with a Tukey's post hoc test.

B. Freshly isolated cardiomyocytes plated in Millicell EZ slide 8-well glass plates (PEZGS0816, Merck Millipore),were fixed with 4% paraformaldehyde-PBS for 15 mins then rinsed 3X with PBS and stored at 4°C. After that, cells were blocked and permeabilized using 3% BSA+ 0.2% Triton-PBS buffer for 30 mins with gentle shaking at RT. The cells were then incubated overnight at 4°C with primary antibodies prepared in the antibody diluent buffer (1% BSA+0.2% Triton-PBS). The next day, the wells were washed 3 times with 3 mins/wash with PBS then incubated with the corresponding secondary antibodies (Alexa Fluor 488 goat anti-mouse (A11029) and Alexa Fluor 647 goat anti rabbit (A21244) : 1/200 - Jackson Immunoresearch) for 45 mins at RT deprived from light. As a final step, cells were washed by PBS again 3 times with 3 mins/wash and mounted with Fluoromount™ Aqueous Mounting Medium (Sigma-F4680). The wells were covered afterwards with coverslip and images were acquired using 60x-objective on confocal microscope (Nikon A1R). The primary antibodies were: mouse anti-GRIM19 (Santa Cruz: sc514111), mouse anti-TOM20 (Santa Cruz: sc17764) for mitochondrial marker, and rabbit anti-HA C29F4 (Cell Signaling #3724S) all used at 1/200 for CypD rescue marker. When cardiomyocytes were not infected (negative control), the red HA staining appears very clear (due to the fluorescence of the cardiomyocyte) as compared to corresponding mitochondria staining visualized with Grim19, where we can see mitochondria network. On the opposite, when cells

were infected by different CypD-HA mutants, we can see in the that the CypD rescue is localized in membrane and into mitochondria stained with TOM20/Grim19. We noticed that sometimes, the TOM20 or HA staining is visualized only in the border of cardiomyocytes. This could be explained by the lack of efficacy of the permeabilization with Triton.

1 Schinzel, A. C. *et al.* Cyclophilin D is a component of mitochondrial permeability transition and mediates neuronal cell death after focal cerebral ischemia. *Proceedings of the National Academy of Sciences of the United States of America* **102**, 12005-12010 (2005).

2 Cabrita, L. D., Dai, W. & Bottomley, S. P. A family of E. coli expression vectors for laboratory scale and high throughput soluble protein production. *BMC Biotechnol* **6**, 12, doi:10.1186/1472-6750-6-12 (2006).

3 Caro-Maldonado, A. & Munoz-Pinedo, C. Dying for Something to Eat: How Cells Respond to Starvation. *The Open Cell Signaling Journal* **3**, 42-51 (2011).

4 Paillard, M. *et al.* Depressing mitochondria-reticulum interactions protects cardiomyocytes from lethal hypoxia-reoxygenation injury. *Circulation* **128**, 1555-1565 (2013).

5 Gomez, L. *et al.* The SR/ER-mitochondria calcium crosstalk is regulated by GSK3beta during reperfusion injury. *Cell Death Differ* **23**, 313-322, doi:10.1038/cdd.2015.101 (2016).

6 Hannich, J. T. *et al.* 1-Deoxydihydroceramide causes anoxic death by impairing chaperonin-mediated protein folding. *Nature Metabolism* **1**, 996-1008, doi:10.1038/s42255-019-0123-y (2019).
