# Supplementary material for: Phosphorylation of cyclophilin D at serine 191 regulates mitochondrial permeability transition pore opening and cell death after ischemia-reperfusion
Source: Cell Death Dis. 2020 Aug 19;11(8):661. doi: 10.1038/s41419-020-02864-5 (PMC7438327; doi:10.1038/s41419-020-02864-5)
Supplement: Supplementary file 2 — Supplemental Tables [file 41419_2020_2864_MOESM2_ESM.docx]

**Supplemental tables**

**Suppl. Table 1**

| **Site-Directed Mutagenesis Primers** | | |
| --- | --- | --- |
| **Oligo** | **Oligo Sequence** | |
| PPIF S38-43A Fwd | gctgctgctgggaacccgctcgtgtacc | |
| PPIF S38-43A Rev | agcagcagccgggtcgccggagccctt | |
| PPIF S119A Fwd | aggcgggaaggccatctacggaa | |
| PPIF S119A Rev | gtgccattgtggttggtgaagtc | |
| PPIF S123A Fwd | catctacggagcccgctttcctgacgag | |
| PPIF S123A Rev | gacttcccgcctgtgcca | |
| PPIF S186A Fwd | gaaaatagaagctttcggctctaagagtgggaggacatcc | |
| PPIF S186A Rev | ttcacgacgtccatgccctctttgacgtgaccgaa | |
| PPIF S191A Fwd | cggctctaaggctgggaggacatc | |
| PPIF S191A Rev | aaagattctattttcttcacgac | |
| PPIF S191E Fwd | cggctctaaggaggggaggacatc | |
| PPIF S191E Rev | aaagattctattttcttcacgac | |
| PPIF R97G Fwd | caccttccacggcgtgatcccttc | |
| PPIF R97G Rev | gagcctttgtagccgaag | |
| **CRISPR Primers** |  | |
| **Oligo** | **Oligo Sequence** | **sgRNA target sequence** |
| PPIF A Fwd | ctcgtgtaccgttttagagctagaaatagcaa | cgggaacccgctcgtgtacc |
| PPIF A Rev | cgggttcccgcggtgtttcgtcctttcc |  |
| PPIF B Fwd | acgggtcgcgttttagagctagaaatagcaa | gaggaagaggacgggtcgc |
| PPIF B Rev | cctcttcctccggtgtttcgtcctttcc |  |
| **Oligo** | **Oligo Sequence** | |
| Backbone Golden Gate Fwd | cgtgtgtggtctcgcaattgtaagcgttaatattttgtt | |
| Backbone Golden Gate Rev | cgtgtgtggtctcgtcattgatgagtttggacaaac | |
| Sg Golden Gate #1 FWD | cgtgtgtggtctcgatgagggcctatttcccatg | |
| Sg Golden Gate #1 Rev | cgtgtgtggtctcgggtacctctagagccatttg | |
| Sg Golden #2 FWD | cgtgtgtggtctcgtaccgagggcctatttcccatg | |
| Sg Golden Gate #2 Rev | cgtgtgtggtctcgattggtacctctagagccatttg | |
| **Sequencing Primers** | | |
| U6 Fwd Seq | ggactatcatatgcttaccgtaacttga | |
| SgRNA Seq Fwd | ttcactgcattctagttgtg | |
| SgRNA Seq Rev | aacgcgaattttaacaaaat | |

List of different site-directed mutagenesis and CRISPR primers.

**Suppl. Table 2**

|  | | Rescue WT | Rescue S191A | Rescue S191E |
| --- | --- | --- | --- | --- |
| Total Injected Mice: | | 14 | 12 | 18 |
| Mice Death | Anesthetic: | 2 | 2 | 0 |
|  | Ischemia: | 0 | 0 | 2 |
|  | Reperfusion: | 2 | 2 | 6 |

Report of deaths that occurred during the anesthetic, ischemia, or reperfusion phase of the I/R surgery for the CypD KO+WT (n=14), CypD KO+S191A (n=12), and CypD KO+S191E (n=18) mice.
