# Supplementary figures and images for: Phosphorylation of cyclophilin D at serine 191 regulates mitochondrial permeability transition pore opening and cell death after ischemia-reperfusion
Source: Cell Death Dis. 2020 Aug 19;11(8):661. doi: 10.1038/s41419-020-02864-5 (PMC7438327; doi:10.1038/s41419-020-02864-5)

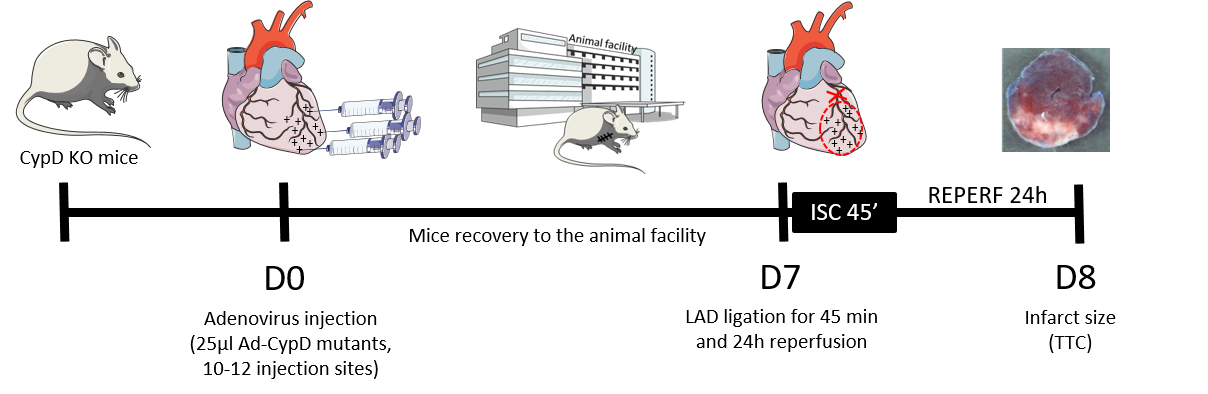

Supplement: Supplementary file 3 — Supplemental Figure 1 [file 41419_2020_2864_MOESM3_ESM.tif]

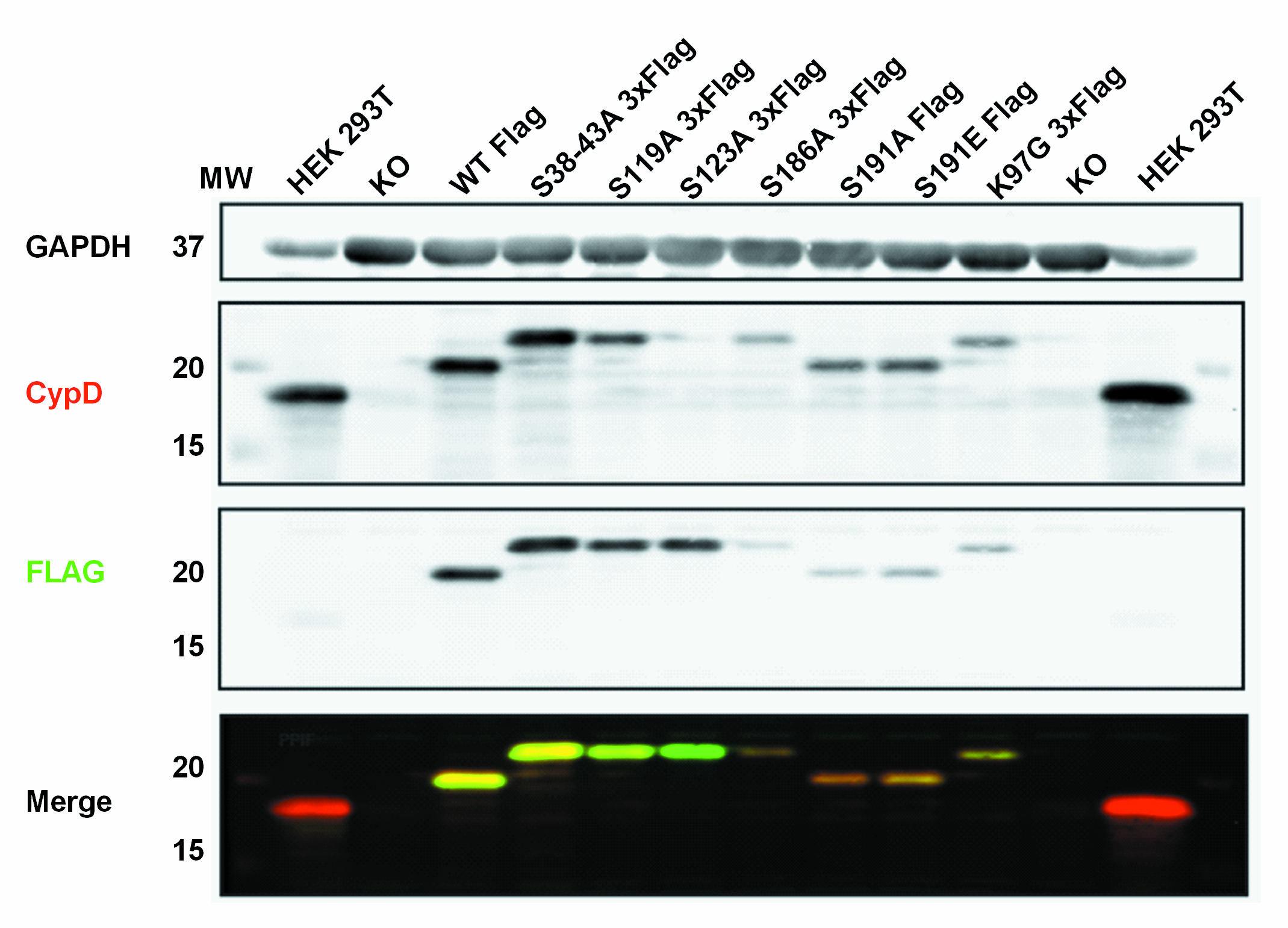

Supplement: Supplementary file 4 — Supplemental Figure 2 [file 41419_2020_2864_MOESM4_ESM.tif]

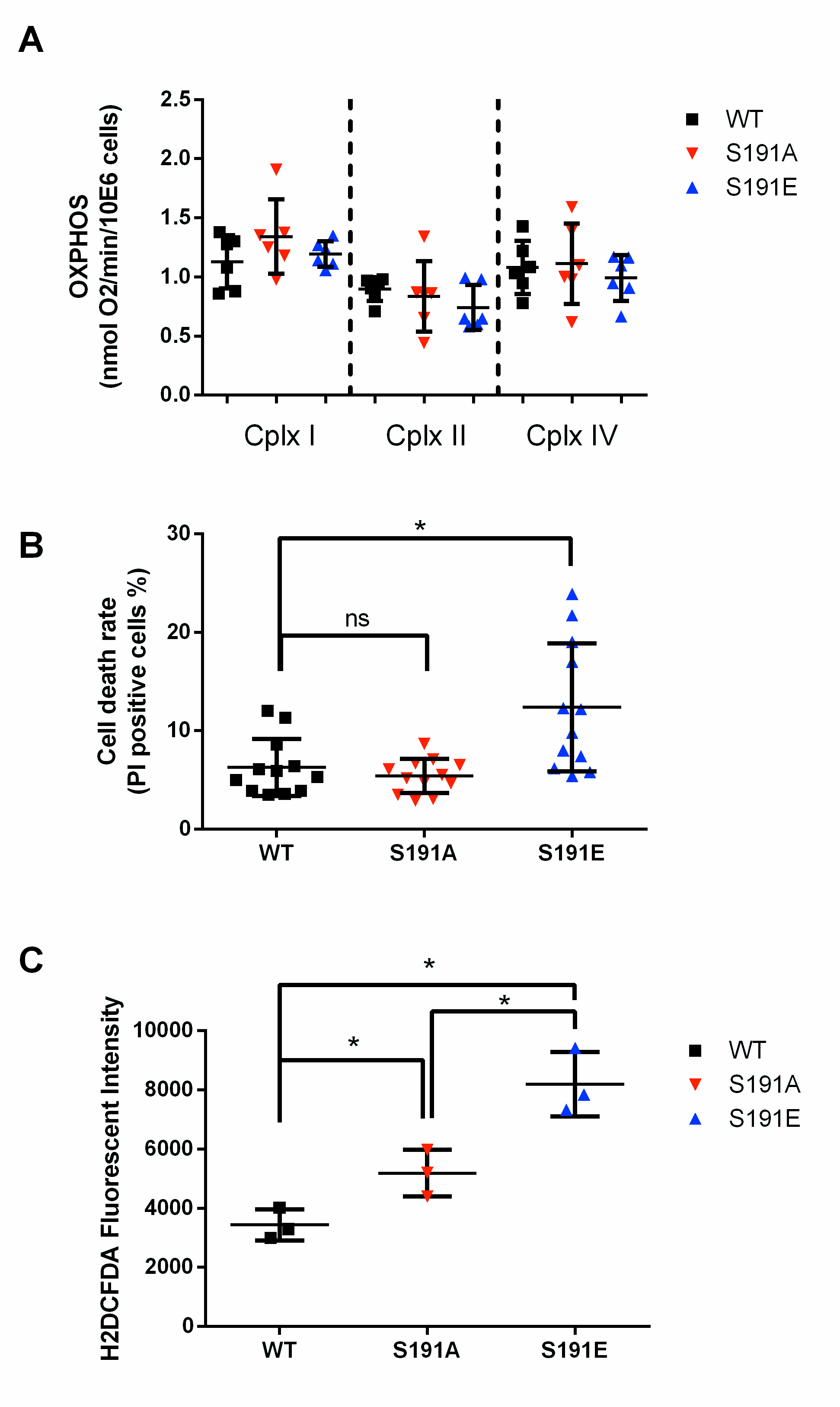

Supplement: Supplementary file 5 — Supplemental Figure 3 [file 41419_2020_2864_MOESM5_ESM.tif]

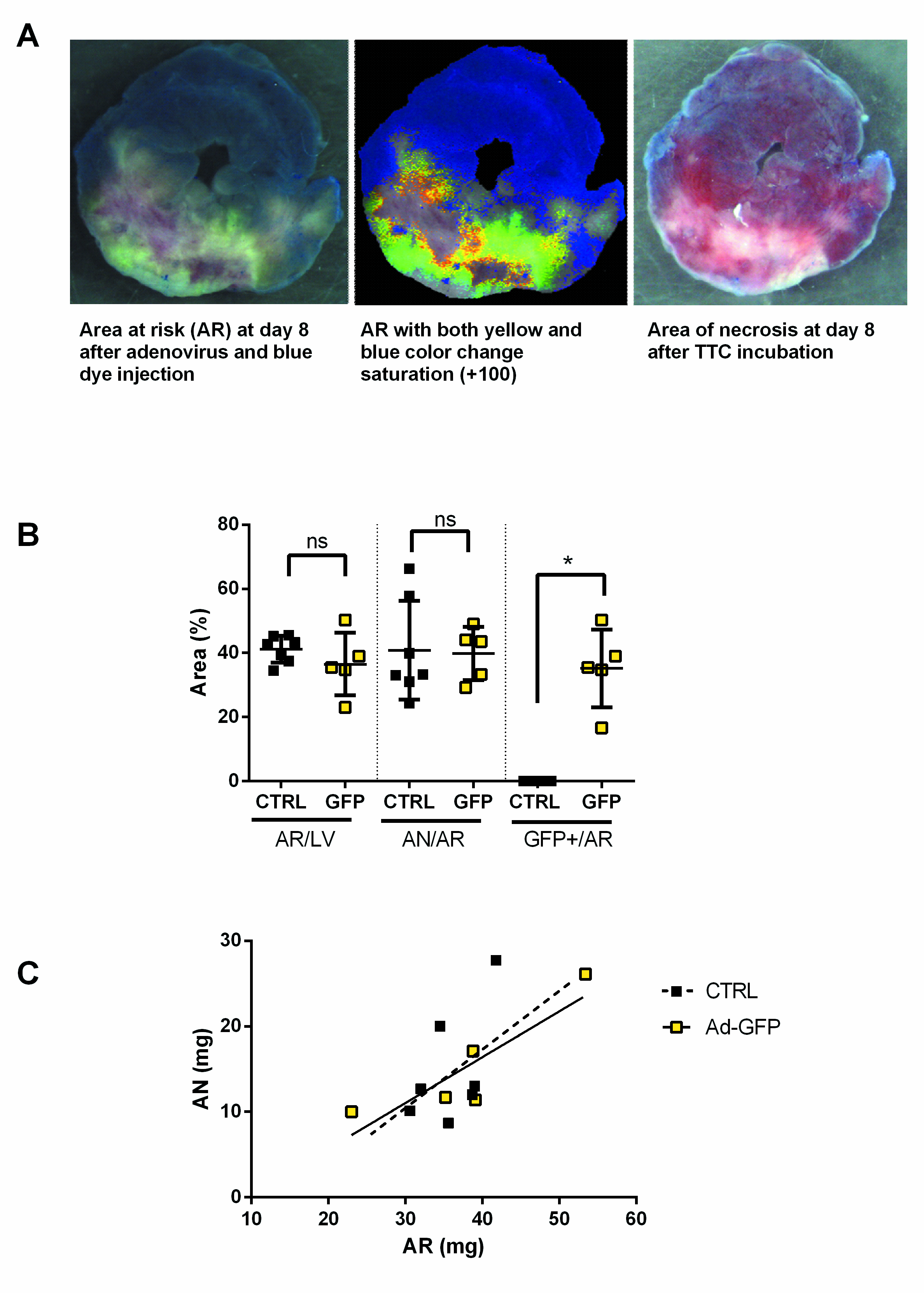

Supplement: Supplementary file 6 — Supplemental Figure 4 [file 41419_2020_2864_MOESM6_ESM.tif]

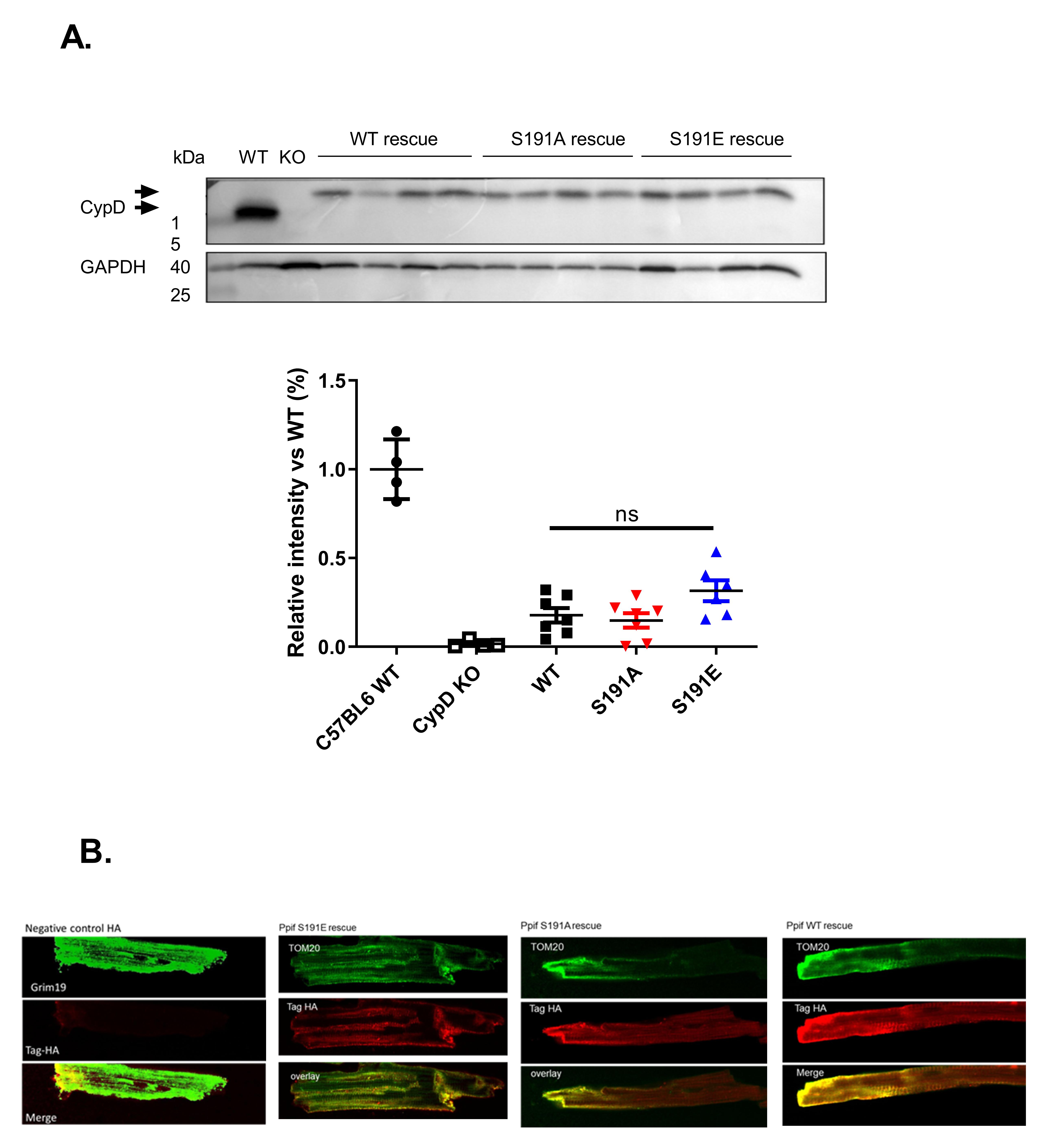

Supplement: Supplementary file 7 — Supplemental Figure 5 [file 41419_2020_2864_MOESM7_ESM.tif]
